# The mattering citizen: young adults with mental illness and complex needs’ experiences and perceived opportunities of social inclusion

**DOI:** 10.3389/fpsyg.2025.1504767

**Published:** 2025-01-29

**Authors:** Silje Nord-Baade, Ottar Ness, Michael Rowe, Camilla Bergsve Jensen, Anne Landheim

**Affiliations:** ^1^Norwegian National Advisory Unit on Concurrent Substance Abuse and Mental Health Disorders, Innlandet Hospital Trust, Hamar, Norway; ^2^Department of Health and Nursing Sciences, University of Inland Norway, Elverum, Norway; ^3^Department of Education and Lifelong Learning, Norwegian University of Science and Technology, Trondheim, Norway; ^4^School of Medicine, Yale University, New Haven, CT, United States

**Keywords:** social inclusion, citizenship, mattering, young adults, social exclusion, mental health, complex needs, flexible assertive community treatment

## Abstract

The negative effects of social exclusion are well known, as are the effects of social inclusion on quality of life and well-being. Young adults with mental illness and complex needs are among the most marginalized people in the community. There is a pressing need to better define and promote social inclusion in mental health and substance use services, addressing both objective and subjective factors. The aim of this qualitative study was to examine the experiences and perceived opportunities of social inclusion among young adults with mental illness and complex needs. This was done to develop a comprehensive understanding of social inclusion that can be applied by providers in the fields of welfare, mental health, and substance use. Seven young adults (three males/four females, aged 22–29) were recruited through Flexible Assertive Community Treatment teams and participated in the study through qualitative semi-structured interviews. The material was analyzed employing an abductive thematic analysis. The findings show the interconnection between the elements of Citizenship and Mattering and underline the need for a framework including both the psychological and sociological perspectives. Developing the Citizenship framework and incorporating the Mattering approach is suggested as a multifaceted approach to promote social inclusion in practice, calling for further research on this.

## Introduction

The increasing rates of young adults who are not in school or employed in Norway is recognized as a major societal problem and is often related to social challenges, mental health, and substance use disorders (Sveinsdottir et al., 2018). Young adults with mental health problems constitute one of the most marginalized group of people in Norwegian society and internationally (Goldblatt et al., 2023; United Nations, 2016).

The people in this group often face challenges related to stigma, interpersonal connections and social reciprocity, vocational engagement, autonomy, unhealthy identities, independent housing and stability (Gardner, 2020; Gardner et al., 2019; Semb et al., 2019; Nord-Baade et al., 2024). In addition, there is a decline in experienced quality of life and faith in the future among younger people compared to older population groups (Goldblatt et al., 2023; Helliwell et al., 2024; Nes et al., 2020; Grimstad and Støren, 2023). The latter may indicate a societal development increasing the risk of exclusion among already marginalized young adults. Furthermore, for young adults in general, the age from 18 through the twenties represents a critical developmental period where striving for autonomy, developing emotional stability, establishing one’s identity, forming adult relationships, establishing a career path and exploring new interests is of particular importance in laying the foundation for a healthy adult life, in addition to learning how to handle the practical aspects (Arnett, 2016). This natural process is challenging for many and additionally complicated for people with mental illness and complex needs.

Social inclusion is often emphasized in promoting mental health and well-being (Kaplan et al., 2012); however, the definition of the term is contested (Denkewicz, 2024; Wright and Stickley, 2013). There are numerous definitions of social inclusion (Filia et al., 2018) and ongoing debates about how the concept should be understood and how social exclusion can be reduced among people in marginalized groups, including among young adults with mental illness and complex needs. The importance of employment, education, housing, neighborhood, social activities and support is most often cited across professional’s definitions of social inclusion (Filia et al., 2018). Some scholars write of the conceptual ambiguity of the term (Rawal, 2008) and on the overemphasis on causes of exclusion rather than paths to inclusion (Sekher and Cârciumaru, 2019). Others write of the need to give greater attention to subjective factors (Gardner et al., 2019; Baumgartner and Burns, 2013) and the importance of employing both objective and subjective factors to gain a multifaceted and comprehensive understanding of social inclusion.

Mental health and substance use services and providers often overlook social factors in treatment and care of people with mental health and substance use problems (Ramon, 2018; Topor et al., 2022; Bjørlykhaug et al., 2022). Research shows a lack of access to person-oriented and integrated care with providers who emphasize and understand people’s social context (Nord-Baade et al., 2024; Ponce et al., 2016). In addition, negative effects of social exclusion are well documented (Goldblatt et al., 2023; United Nations, 2016; Brandt et al., 2022). For young adults with mental illness and complex needs it is crucial to address this issue, as they are one of the most marginalized groups in our communities (Goldblatt et al., 2023). There is a need to develop and communicate the concept of social inclusion to promote it.

### Theoretical framework

The Citizenship framework operationalizes the concept of social inclusion through the 5 Rs of rights, responsibilities, roles, resources, and relationships that society offers its members through public and social institutions and a sense of validated belonging (Rowe, 2015). The R of *rights* ensures that individuals have access to their legal and human rights, including the right to fair treatment, privacy, and participation in society. *Responsibilities* are personal responsibilities as well as civic duties, and a responsibility both toward oneself and fellow citizens. Valued *roles* involve supporting individuals in finding and maintaining valued roles in society, whether through employment, volunteering, or other forms of valued roles, such as being a parent or a friend. *Resources* include access to necessary resources such as healthcare, education, housing, and social services. In addition, it includes personal resources such as the person’s gifts and capabilities, and of persons who provide social support. The fifth R involves positive *relationships* with family, friends, the community, and systems, from personal relationships to the relationship one has to the larger community. In addition to the 5 Rs, Citizenship also includes a sense of *belonging* in society that must be *validated* by others. Rowe’s model aims to promote social inclusion and empower individuals with mental health and substance use problems to create and lead fulfilling lives in their communities.

While Citizenship is a promising framework in practice, it does not directly address subjective factors within the individual. Such subjective elements may be the fundamental sense, or lack of it, of being someone of value to others, or having something to offer. Additionally, the framework does not directly address people’s negative self-perception and identity, important barriers to social inclusion for people who are marginalized (Nord-Baade et al., 2024; Kidd and Davidson, 2007; Liljeholm and Bejerholm, 2020; Storm et al., 2023; Thulien et al., 2019). These subjective factors, however, may improve because of positive developments in the areas that the 5 Rs covers. The sense of validated belonging is based more on subjective perceptions, but the source of it is external feedback through the validation that community offers the persons. One strength of the model is that it offers a tangible approach, providing a language and guidance as to how to approach the promotion of social inclusion. Another strength is the strong user involvement in development of the framework (Rowe et al., 2012). Further, it places parts of the responsibility on other members in the community and the systems.

On the other hand, though, people who are provided with the 5 Rs and the validated belonging may have difficulties adjusting to their new situations, hence returning to old ways of life (Rowe, 2015; Thulien et al., 2019). This may suggest the need for something in addition to what the Citizenship framework offers. Research shows that social inclusion is related to the fundamental and interconnected needs of being someone of value and having something of value to offer to others and of being able to contribute and make a difference in the world (Nord-Baade et al., 2024; Flett, 2022; Paradisi et al., 2024; Prilleltensky, 2020).

The theory of mattering addresses wellbeing and social inclusion through people’s sense of feeling that they do or do not matter to themselves and others (Prilleltensky and Prilleltensky, 2021; Prilleltensky et al., 2023). This is strongly connected to psychological traits such as motivation, meaning of life, a sense of self-efficacy and perceptions of fairness (Prilleltensky, 2020; Prilleltensky et al., 2023). Furthermore, mattering is also connected to conditions of justice (Prilleltensky, 2012). Still, mattering moves beyond the mere objectively recognized aspects of having value or adding value. It is the psychological experience of feeling valued or adding value through the four domains of self, relationships, work or main occupation, and community. The balance between adding and feeling valued is a key element. Just as important is the balance between the domains mentioned above with self and others as sources and beneficiaries. Though overlapping on some parts, the mattering framework might be a helpful way of directly addressing the subjective features of social inclusion creating synergistic effects when seen together with citizenship’s 5 Rs and validated belonging.

### Research objectives

Developing a framework consisting of citizenship, mattering and a narrative identity approach (Nord-Baade et al., 2024; Arnett, 2015; Birney, 2023; McLean and Syed, 2015; Nord-Baade and Rowe, 2023), might be a way to promote social inclusion through a more comprehensive understanding than each of these concepts offers alone. Drawing on social constructionist (Gergen, 2022) and participatory methodology approaches (Beresford, 2013), and building on previous research on identity and social inclusion among young adults with mental illness and complex needs (Nord-Baade et al., 2024; Kidd and Davidson, 2007; Liljeholm and Bejerholm, 2020; Storm et al., 2023; Thulien et al., 2019), the aim of this qualitative study is to examine the experiences and perceived opportunities of social inclusion among young adults with mental illness and complex needs. We do so by employing the Citizenship framework (Rowe, 2015; Rowe, 1999; Rowe, 2017) followed by discussing the potential to combine Citizenship with the Mattering approach. By doing so we seek to contribute to a multifaceted understanding and concept of social inclusion. This can be employed in promoting social inclusion in practice by providers in welfare, mental health and substance use services.

## Materials and methods

### Design and paradigm

The social constructionist approach considers knowledge as co-created and context sensitive (Gergen, 2022). It views experience as a combination of personal factors and social influences and of the dynamics between them. A participatory design was chosen to ensure relevance, validity and accountability, and to acknowledge the value of combining lived experience and academic knowledge (Beresford, 2013). Employing a participatory design in a social constructionism paradigm is especially relevant when addressing complex social problems.

The relevance of studying promotion of social inclusion among young adults in the target group was discussed with different experts in the field and people with lived experience. Among people with lived experience were two young adults with extensive lived experience of mental health problems, whom at the time were members of the national advisory board on the implementation of Youth FACT in Norway. In addition, a peer researcher (fourth author) actively participated in the research process, from planning to the dissemination of findings. Therefore, participation occurred at two levels; consultative and participatory (Beresford, 2013).

### Research context

Participants were receiving services from FACT teams (Flexible Assertive Community Treatment) and recruited to the study through those teams. FACT is a multidisciplinary service model providing integrated care for people with severe mental health and/or substance use disorders (van Veldhuizen, 2007). FACT represents a positive development in Norwegian services that struggle to provide integrated care for this group (Landheim and Odden, 2020; Nord-Baade et al., 2022). In Norway, the teams are a collaboration between providers in the specialist mental health care and the municipal health services, drawing on expertise from both levels of care and peer support. Social inclusion is an integral part of the model, with provision of services in the local community and potential access to employment, education, meaningful activities, or through including family, social network and the neighborhood in treatment and follow-up. Three FACT teams were included in the study: a rural team, a semi-rural and a team from an urban area, all in Eastern parts of Norway. Three teams were included partly because of recruitment challenges, however, including young adults from different areas may contribute to understanding how social inclusion is experienced in different areas, important perspectives as supported by, e.g., intersectionality theory (Cooper, 2016). The study is part of a larger project investigating promotion of social inclusion among young adults in FACT teams.

### Recruitment and participants

Team staff were told about the study and contributed to the recruitment process. All patients between the age of 18–30 were eligible for recruitment. Those interested were given additional information and a consent form. Staff excluded some patients due to high symptom levels at the time. Patients who consented to participate were contacted by the first author and interviews were scheduled. As the interviews provided rich material with acceptable information power (Malterud et al., 2016), seven participants were considered sufficient.

Seven participants (four women and three men) participated in the study. Their ages ranged from 22 to 29 years (Mean = 26). All participants were born in Norway, none had immigrant backgrounds. Two participants lived in urban regions, two in semi-rural, and three in rural regions. They spoke of receiving services from FACT teams mainly due to anxiety, depression, psychotic, or neurodevelopmental disorders. Most reported having more than one mental health disorder and impaired everyday function. One participant disclosed present substance use and three spoke of past substance abuse, both illegal substances and alcohol. One participant reported having a job; the others had extensive histories of failing to keep or obtain work. Four had dropped out of school before or during high school. Two had tried to attain a degree in higher education but dropped out. Three of the participants had histories of violence with two reporting extensive criminal histories. One was currently in a substance use treatment facility, another was serving time in prison, and another participant had previously been imprisoned.

We used the pseudonyms Sophia, Mary, James (participants in rural areas), Jennifer, Peter (participants in semi-urban areas), and Catherine, Peter, and William (participants in urban areas).

### Data collection

Individual semi-structured interviews were used to collect data to enhance opportunities of in-depth understanding of participants’ subjective experiences through a flexible exploration (Denzin et al., 2023). Another reason for choosing individual interviews was the need to create a safe environment for a group of vulnerable people. The interview guide contained open and general questions on perceptions and experiences of social inclusion and exclusion, as well as specific questions related to citizenship, mattering and identity. All authors contributed to the development of the interview guide. Through this process, discussions between authors included critical reflection on biases and assumptions.

Out of seven participants, four were interviewed in person, two by telephone and one by online video interview. Two participants preferred being interviewed by telephone and online interviews, whereas the interview with the incarcerated participant had to be conducted by phone. The first author conducted the interviews, aiming to explore participants’ experiences of social inclusion and ways to promote it for people like themselves. Participants were also asked about their experiences with the five Rs of Citizenship, their sense of mattering, self-perception and identity, and asked their views on people in their local communities and perceptions of their communities in general. In addition, they were asked about their experiences and views on how FACT promotes social inclusion and how they should address this matter. Participants were given the opportunity to provide additional statements at the end of the interview. None did. The length of interviews was 30 to 77 min, with an average duration of 56 min. Interviews were recorded and transcribed verbatim by the fourth and first authors.

### Analysis

An abductive thematic analysis approach (van Hulst and Visser, 2024; Braun and Clarke, 2021a; Braun and Clarke, 2021b) was chosen. Using thematic analysis enabled us to give voice to the participants (Braun and Clarke, 2021a), ensuring that these perspectives and experiences were in focus. This approach aligns with the social constructionist emphasis on the co-construction of meaning. The aim of the study was to explore and make meaning of the experiences of social inclusion and perceived opportunities through an already existing framework. Furthermore, this was done with the intent of developing it through exploring important subjective perspectives that appeared to be missing in the framework.

The first author conducted the analysis; however, all contributing authors were consulted throughout the process. In phase 1 the material was read, re-read, and discussed, familiarizing all authors with the data. In addition, the peer researcher had a special role in the preparation phase through transcribing and reading the material, providing independently written inductive-driven summaries from each interview and suggestions for next steps of the analysis. These suggestions created an important foundation for the initial coding and generation of themes. The first author proceeded with the second step of analysis, with inductive-driven initial generation of codes (phase 2). Following this, the analysis proceeded with generation of initial themes (phase 3), then reviewed the themes (phase 4), defined and named them (phase 5). In these latter phases, the material was organized according to the Citizenship framework with subcodes emphasizing subjective interpretations and connections, guided by the answers to the open-ended questions and the mattering perspective. There was some movement back and forth between the different phases following discussions of the material as the understanding of the material continued to evolve and by comparison to previous research, before writing it up (phase 6).

Critical awareness and reflexivity was exercised to avoid biases in collection and interpretation of the data, through re-reading the material, re-examining the findings, and through discussions during the analytic process (Finlay, 2002).

### Ethical considerations

The study was approved by the local data protection officer at Innlandet Hospital Trust (ID 22587532), following recommendations by the Regional Committee for Medical and Health Research Ethics in Norway (ref no. 482463). Participation in the study was consent-based. The consent form contained information on confidentiality, that participation would have no impact on the services they received, and that they could withdraw from the study at any point. This was repeated orally before the interviews started to ensure informed consent. Participants were reassured about the value of their contributions and sharing their experiences. They were given the chance to ask questions or voice their concerns before the interviews started. None did. At the end of the interview, the researcher asked about the participant’s experience in the interview situation to ensure that no harm was inflicted. Participants received a gift card of 500 NOK for their contribution. There were no signs of this influencing the integrity of the research.

## Findings

Participants spoke of having limited awareness of the concept of social inclusion. This is interesting considering that nearly all experienced major feelings of outsiderness and feeling labeled as outsiders by the mainstream community. Further, nearly all were living lives with limited community participation and contact with others, with some actively withdrawing from social arenas. As the interviews progressed, and their knowledge of social inclusion increased, most participants expressed interest in the concept and the importance of it. They had few experiences of talking to welfare or health providers about who they wanted to be in a social context, or of talking about social inclusion as part of and possible outcome of treatment. Strikingly, inherent in their experiences of social exclusion and their wish to be included, was the notion of not mattering and wanting to matter. On the other hand, they spoke of elements of social inclusion that were integrated in their treatment or accessible, with activities and employment most frequently mentioned. This could imply that social inclusion is largely understood and addressed through these means by welfare and mental health providers.

The findings are presented in relation to the 5 Rs of Citizenship framework following the abductive thematic analysis.

### Rights

All participants were currently receiving services from a FACT team and had previous experience of being in contact with other parts of the health and welfare system. In addition, all had challenging histories related to their school years, and some had extensive histories with child protective services and the criminal justice system. In sum, they all depended on knowing and practicing rights from early on. Participants who dropped out before or during high school told stories of not receiving help even when they showed clear signs of academic or social difficulties, despite being entitled to accommodations by law. They spoke similarly about their histories, with several losing their jobs in part through not knowing of, asking for, or being offered support that could have helped them master employment. As part of their treatment, most of the participants expressed knowledge of and practiced their rights of shared decision making and user involvement, feeling respected and heard. However, some said they did not know if they were aware of their rights in terms of access to services or means that could help them. James said,

“They have always said that I am the main focus and that I need to speak up about what I need. But I find it difficult to demand anything. And I do not really know what it takes for things to work for me either. I feel like I have not received that many suggestions either.”

James further explained that he had told welfare and mental health providers about this insecurity but experienced getting little response. Participants often struggled to speak up about their needs and did not know their rights. In addition to this, some had difficulties practicing their rights when they needed something else or more than what they were offered. Even so, they said they received satisfactory treatment in general and had developed a good relationship with the team. Sophia, after not receiving the treatment she was promised, said,

“And then I’m a bit hesitant to speak up, like what happened with that, were we supposed to do that, eh, I’m afraid that they’ll think ‘no, it’s just no use… I am generally like that, so it does not really have anything to do with the team, but it’s just that you feel a bit pushy, maybe, you feel like, at least I am like that, that I feel like I’m nagging, and I feel almost even sicker if I start to nag.”

This points at a connection between practicing one’s rights, self-perception and sense of value that goes beyond having a satisfactory relationship with the providers. A sense of not mattering seemed to function as a barrier to ask for necessary information and practice one’s rights. Further, the participants’ histories of not always being heard or treated as entitled to appeared to influence their sense of not mattering in a system’s perspective.

### Responsibilities

The participants spoke of wanting responsibilities, of wanting to contribute, but of not being given the chance or feeling able to do so at that time. Some doubted they could lead ‘normal lives’ with the responsibilities of work, family, friendships, finances and housing. Participants knew they had responsibilities toward others, and several talked of supporting their friends and family. They knew they had to take care of themselves and to build their own lives, but experienced varying levels of confidence and expectations from others. Connected to a sense of mattering, the participants experienced being labeled through perceived messages from the mainstream community, and partly by the services, as less of value due to the limited amounts of responsibility they were either able to handle or offered. This was to a large degree internalized.

The R of rights is connected to the R of responsibilities. Sophia talking about the right of user involvement, but at the same time she said,

“I get the feeling that I am the boss, but I need, I am very dependent on that kick in the butt as well […]. I know there are many who have it [accommodations at work],” she said, “it’s just that you do not hear about it. And when it comes to discussions, like now, when I’ve talked to a career advisor, it’s never been an option. It’s only talk about ‘yes, you’ll probably be in full-time work in a couple of years and things like that. And it’s never been discussed that we could start with maybe just a couple of hours once a week. So that’s how I feel, I do not know what kind of opportunities I have in a way. Of course, I could ask, but then there’s this thing about hesitating to ask about such things because you do not know if it’s even possible.”

Sophia’s comments illustrate the importance of being given a chance to have responsibilities that are adapted to one’s needs and capabilities. Further, it shows the service providers’ roles in helping young adults to take responsibilities by both providing them with information about relevant means and supporting the level of responsibility that is manageable for each person. Others had both similar and opposite experiences to Sophia’s. Jennifer said,

“I have been in the system for a long time. They have tried many different things. There has been a lot of pressure to get out into work, which is very understandable since I was very young when I entered the system, but now I am on disability. Now they are almost completely quiet, I’m really just focusing on my own health now.”

Jennifer’s experience appears similar to that of Sophia, representing the same “all or nothing” approach regarding work. Another interviewee, James, spoke of a combination of low self-esteem, increasing demands and responsibilities, and the pressure he felt as he grew older, leading to him pretending to have everything in order. This further underlines the importance of providers communicating accommodated levels of responsibility and that they recognize and validate the value that lie in what each person can offer, even if it is limited compared to perceived expectations and standards.

### Roles

As with responsibilities, the participants spoke of having few valued roles and were doubtful as to whether they were able to acquire such roles and add value through these. Valued roles were mostly described as being someone with a family, a social network, and someone who had a job. However, the roles of being a patient, someone with none or few friends, unemployed or uneducated, someone who did not contribute to the community, and someone who received benefits were dominating perceived roles in most participants. This sense of uselessness influenced their perceptions of mattering or not mattering. Peter said, “I just sit at home watching YouTube.”

Still, a few of the participants described having some valued roles, such as through helping others, doing voluntary work, or by being in an adapted work program. James, who currently was part of a work program, said this,

“It made a big difference. Now I dare to be a bit social because I can say that I have a job […]. I am not sitting at home twiddling my thumbs and taking money from the state.”

He further explained that he presented it as having a regular job to people he met out of fear of negative feedback and shame, revealing his understanding of the perceived external value of his contributions and fear of devaluation. Catherine spoke of being in the process of changing and acquiring new and valued roles,

“I do not think much about the past because I’m no longer an active drug user. I have put that behind me, and it’s not something I carry with me into the future. Now it’s more about working on things like finishing my education, wanting to start a business, and my love for animals. More positive things like that.”

Still, most of the participants were uncertain as to how they could attain valued roles, and as important, if they were suited for them or ever could be. This was demonstrated by Mary who spoke of losing faith in herself because she recently had to quit her job, Peter who saw himself as “a loser,” and Jennifer who experienced more hopelessness and self-hatred when comparing her life with others in terms of not having the same valued roles. Having valued roles represents important sources to experiencing mattering. As evident in the material, the participants’ experiences of feeling as they did not matter yet again seemed to create hindrance to acquiring valued roles, and leaving unvalued roles behind.

### Resources

As previously mentioned, all participants had histories of receiving help from services and providers which one could safely assume had either been exposed to or given training in recovery-oriented practices. Inherent in this is the focus on mapping and empowering individuals through their personal resources. Strikingly, most participants found it difficult to name personal resources and expressed uncertainties whether they had valuable personal resources or not. Asked if service providers address the subject, Sophia said,

“I do not think I’ve ever really been asked that question, like how do you think, or what kind of resources do you feel you have, in a way, I do not think I’ve actually been asked that.”

Mary was uncertain and stated, “It might well be that we have [talked about resources], it’s just that I do not remember.” Others remembered having such a conversation, but several had difficulties with providing examples of what their personal resources were. Peter pointed at another factor, “I do not feel like doing anything, that’s the thing. It’s because of the medications,” further stating that he might have some resources, but was unable to use them practically. The participants also spoke of low levels of education or histories of dropping out of school, and not mastering work, as previously mentioned, with this often coloring the view on their personal resources. The connection between not knowing or validating one’s resources might be connected to not experiencing that their resources were desirable or of value, as previously noted in relation to the R’s of responsibilities and valued roles.

On having resources in terms of access to treatment or welfare services, all participants had negative experiences, especially with previous encounters. Catherine, who had a long history involving the child protective services, the criminal justice system, welfare services, and mental health and substance use services, pointed at a perceived gamble in this contact,

“It’s very difficult to be a part of the system in that sense. Because you can meet one of two people in the system, people who genuinely want to help, or the exact opposite. It’s never a middle ground, I feel, and I also hear many others say this. Many people have that experience.”

Still, in terms of housing and receiving help with sorting out finances, most reported that they had access to this form of resource. However, financial resources were scarce, and participants saw this as a barrier to engaging and participating in their local communities. As modern society value material resources, one can assume that these aspects are other sources of feeling insignificant with little of value to offer.

### Relationships

Participants’ relationships with their families were most frequently mentioned when asked about the R of relationships. Most had present or past challenging relationships with their families, but some reported the opposite, like Jennifer, “My best friend is my little sister, and my mom is someone who has always been there. […] They are my closest ones.” William spoke of being welcomed back to his family, “When I was using drugs, I felt excluded, but now that I am sober, they have welcomed me back.”

Several participants spoke of having none or few friends, and some spoke of having lost contact with friends or acquaintances, both involuntary and by choice. James found that his friends had let him down and cut off contact with him because of his struggles,

“I explained how I was feeling when I was admitted to the acute psychiatric unit, said that I was really struggling and that I could not be as social right now, but that I still wanted to be invited to things, to see that they still wanted to be friends with me. But then all my friends disappeared, and I was left alone again.”

He believed this was connected to stigma and lack of knowledge,

“I think they were afraid of doing something wrong and just wanted to leave me alone. They think it’s better to do nothing. […] So, they backed away.”

Catherine had similar experiences dating back to when she was younger,

“When it gets out that you are under child protective services, it’s a bit like, there are a lot of people who do not want anything to do with you, because of the reputation you get. I feel that there is a lot of misinformation among people. I have lost contact with quite a few.”

The fear of losing friends if they said too much about their struggles was apparent in other participants. Sophia spoke of having two friends, but none knew about her long history with severe struggles. William said this about losing friends: “I have lost quite a few friends. At least I have called them friends. They have passed away because of drugs. And people keep passing away. […] I think I am up to about 15–16 people.” Others told about cutting off people that had a negative influence on their recovery, like Catherine who had few friends left, “I have cut off contact with quite a few people. You end up sitting around without much to do really” further explaining how loneliness was an equal negative influence,

“We have group therapy here [unit for substance use treatment], […] all the others also speak about loneliness being a very big trigger making people go back to basically everything that is bad. I would say that such things [loneliness and losing friends] can steal your whole life.”

All but one participant, who had a large group of friends, spoke of wanting to have healthy relationships and a circle of friends. James spoke of needing reassurance and feeling insecure,

“Since I have such low self-esteem, I need to see that people really want to include me in something. I’m afraid of being pushy and imposing, and I’m afraid that they invite me along just because they feel sorry for me and not because they like me.”

Peter had similar experiences, and said,

“Well, I do have some friends, of course, but it’s always up to me to start a conversation with them. It’s always me who calls, it’s always me who sends a message. So that’s kind of a bummer actually.”

Peter also spoke of insecurities in the actual social situation, “I’m one year drug-free, but alcohol, I’m still struggling with that. It’s one thing I actually do not want to quit. It would have been social suicide, quite simply,” clearly showing the need for strategies when socializing.

These direct messages of losing personal value often due to struggling in various ways had clear implications for developing a sense of not mattering, and a fear of not having anything of value to offer in the search for new relationships. As with the negative roles, the effect of stigma was evident and largely internalized.

### A sense of validated belonging

In general, the participants talked about lacking a sense of belonging to the mainstream community, feeling like outsiders for large parts of their lives. Their added and previous experiences of failing to meet perceived standards, and for some feeling poorly treated by society, left them feeling insecure as to if they ever could truly experience a validated sense of belonging. Some were uncertain about whether they wanted to belong to the mainstream community. This appeared connected to perceptions of local community members as individualistic oriented and their self-perception of not fitting in or having little to offer. Most felt they did not matter in society as a whole in turn affecting their conflicted feelings toward belonging. However, nearly all participants spoke of finding a sense of belonging in some areas, but often spoke of this accompanied by feelings of grief and unfairness, due to thinking that they were missing out on something which they perceived that “everyone else” was experiencing.

Catherine, who had grown up in foster care and institutions, stated that she had never felt a sense of belonging. She nowspoke of feeling that she felt a belonging in the spiritual alternative community where she finally had found answers to lifelong questions and wondering. She connected the sense of belonging to contributing and feeling validated through this group:

“Those I have tried to help say that it has actually helped and that they can move forward with things. So, I feel that I have something to contribute with, and that is a sense of belonging.”

Later, referring to conflicts in different parts of the world and how people in need, including herself, were treated by the health and welfare systems, Catherine spoke of distancing herself from the mainstream community as a protest. This shows how societal development directly influence social inclusion and a sense of not mattering for some:

“I simply feel that I do not agree with much of it, and that also makes me consciously choose to distance myself from society. Because much of it is things I simply do not agree with or accept.”

Others who experienced feeling distant to people in the mainstream community, found a sense of belonging online through social media or gaming. These areas felt more right for them, as they could speak more freely about who they were and what they struggled with, and connect with others like them who gave validating feedback. In this way, social media gave them a break from what they perceived as negative and difficult in mainstream community participation. Further, a sense of mattering was more easily achieved.

Others found that social media only heightened their sense of not mattering as it was a major source to feeling inferior, given the picture-perfect images of people portraying their place in society and among others. Sophia pointed at a general perceived pressure to be perfect to be able to experience a validated sense of belonging, and said,

“In many ways it [the pressure] can become very overwhelming, to the point where we [young adults with mental illness and complex problems] eventually give up. That we think that the goal is so far away that it’s not even worth trying anymore. I would think that there is a lot of that.”

Some highlighted the role of family and friends, but not all experienced a validated belonging through them; rather, they felt like outsiders, and invalidated. James, who always had felt he had to hide his struggles from his family and previous colleagues at work, spoke of feeling like he had to hide his thoughts and emotions to experience a sense of belonging. Mary spoke of a sense of belonging through her family and two friends, although later in the interview she said her friends did not know how much she struggled and that this made her feel lonely. William, who did not feel as though he belonged anywhere, said he distanced himself from family and friends to protect them and keep them safe from problems associated with his past drug use and criminality. Jennifer said she felt belonging through her family and friends but also through years of voluntary work at sports events with a local sports team, a place where she felt safe. Peter, with a history of violence, lost his friends, with whom he had a sense of belonging. As apparent in the material, it is difficult to experience a sense of mattering if you do not belong, and vica versa.

## Discussion

The aim of the study was to examine the experiences and perceived opportunities of social inclusion among young adults with mental illness and complex problems. This was done through employing the Citizenship framework to investigate the potential in developing and integrating elements of Mattering. The overarching purpose of the study was to address the need for a multifaceted and tangible approach to social inclusion, highlighting both objective and subjective factors through these frameworks. A sense of being worthless and not mattering is a commonly known phenomena among people with mental illness not only to specific symptoms but also as a result of life experiences (Flett, 2021).

Our study confirms previous findings on challenges that this group often faces (Gardner, 2020; Gardner et al., 2019; Nord-Baade et al., 2024). Further, it proposes, based on its findings, that service providers need to focus on their clients’ social contexts. The concept of social inclusion and the benefits of it is under communicated to the target group. The participants spoke of some elements of social inclusion being addressed by service providers but reported few means. Furthermore, they recalled none or few conversations addressing social inclusion directly. Dialog is essential for fostering a sense of inclusion and mattering. By actively listening and validating the person’s experiences, the service provider can build trust and empathy, making the patient feel understood and valued (Ljungberg et al., 2015). Open communication can raise awareness, encourage and motivate the person to promote their citizenship and sense of mattering, enhancing a sense of agency and overall well-being. There is a need to develop a holistic, multifaceted and tangible approach to promote social inclusion among people in this marginalized group. This includes developing a common language on these subjects facilitating the dialog as described above.

Past influences on feeling excluded and not mattering appeared across common grounds such as family, school and early work experiences. Across rural and more urban areas, the participants appeared to have a similar state of mind related to not fitting in. The demographic and social context did not appear to influence the participants’ understanding of the concept of social inclusion but had some impact on perceived opportunities for social inclusion as rural areas were perceived to offer fewer opportunities and as less attractive.

Further, compared to experiences with mainstream community, an equal source of feeling as they did not matter and that they were excluded, or the opposite, appeared to come from social media where they either connected with peers or experienced not fitting in once again. This may suggest a partly shift of attention to seeking mattering and citizenship in the digital communities, leaving the mainstream community somewhat less prioritized. This may have negative implications on traditional efforts to promote social inclusion and should be further explored. Online communities can serve as support networks for young adults with mental illness and complex needs, offering a sense of belonging and understanding that might be difficult to find elsewhere. The accessibility of social media platforms allows individuals who struggle with face-to-face interactions to engage and connect with others, promoting social inclusion. Further, online platforms can empower young adults with mental illness and complex needs by giving them a voice and agency to share their experiences and advocate for their rights. However, it’s important to balance online interactions with real-world social interactions to prevent isolation and ensure comprehensive social inclusion.

The psychological, subjective sense of mattering approach may be seen as complementing the citizenship framework with its emphasis on social inclusion. Further, our study suggests that Mattering may be connected to the young adults’ engagement required to do Citizenship work, enhancing access to the 5 Rs and validated belonging, in turn reinforcing a sense of Mattering. The opposite may also be true - that lack of engagement with the community may reinforce the feeling of not being of value. Citizenship and Mattering are not seen as inherent but created and maintained through the interactions in the individual’s social context, in line with social constructionism. It is our view that understanding these processes in relation to social inclusion calls for a framework including both the psychological and sociological perspectives.

### The connection between citizenship and mattering

Statements from our study participants suggest that combining Citizenship and Mattering can be a productive approach. This is based on the lack of value they assigned to themselves and their contributions, and further, how this was inherent in the way they spoke of their experiences and perceived opportunities of social inclusion when addressing the key elements of Citizenship. The findings show different layers of social inclusion, in which participants are aware of the importance of the 5 Rs and the validated belonging, but unable to achieve them, since the sense of not mattering is a barrier to achieving the 5 Rs and validated belonging. Study participants often perceive themselves as being people of less value and losing because of this, thus hindering them in doing ‘citizenship work’. This understanding is in line with the capability approach (Nussbaum, 2003; Nussbaum, 2011), pointing out the complexity in empowering marginalized individuals, and the need for a multifaceted approach combining both objective and subjective means.

Regarding rights, participants spoke of not practicing them due to a lack of ability or confidence to demand what they were entitled to, or lack of knowledge of what they could expect. They shared stories of not receiving help despite well-known treatment guidelines and legislative rights, and of negative experiences in the school system. It seems plausible that they perceive themselves as less valuable because of previous systemic failures and that these experiences have led to a passive stance that hinders from demanding to be treated fairly, in the adding value to themselves. This underlines previous studies showing that collaborative practices with practitioners must involve practical help (Ness et al., 2017) and assistance in matters regarding their rights. Failing to meet this need may further build upon their sense of not mattering, further undermining efforts of social inclusion.

The capability approach (Nussbaum, 2003; Nussbaum, 2011; Sen, 2005; Ness et al., 2020) also supports the findings in terms of responsibilities. The participants spoke of wanting to have more in regard to others and society but were stymied in part because of not working. They were overwhelmed by both a perceived pressure to perform and personal insecurities that made them question the value of the responsibilities they managed. Further, some spoke of a perceived “all or nothing” attitude from employers, welfare services, and society. For those who considered working a few hours or days a week, this led to a feeling that their contribution was not sufficiently valuable, thus hindering them taking on new and more robust responsibilities. One’s perceived low expectations confirm their sense of their lack of value. Previous research shows the importance of not being given up on, and points at the essential experience of having someone believe that one is capable of contributing in order to re-establish a sense of value (Ness et al., 2017; Deegan, 1993). This is also connected to the R of roles, as discussed below.

Participants’ sense of value seemed to be confirmed by their current roles, which were largely not of value to them. It became evident that participants could add value to themselves and others through contributions in addition to employment, such as being a good friend or being in an adapted work program. This implies nuancing their sense and sources of value, exploring more sources and widening the perceptions of what is perceived as valuable. It is important that clinicians and providers help build upon this. As previous research shows, participating in activities with adapted levels of personal responsibility and engagement can support the process of acquiring valued roles through a step by step approach (Bjørlykhaug et al., 2022; Nord-Baade et al., 2024), parallel to building upon a sense of mattering.

It was striking that people did not talk about personal resources, as this is a fundamental part of recovery-oriented services (Slade, 2009) such as FACT teams. The dominating self-perception of not being someone of value seemed to be at work in this area. Obviously, the participants had personal resources, but there was a systematic undermining of these resources, either by not naming any or by comparing them with other people’s resources. Not knowing your personal resources, and therefore not building on them, will inevitably contribute to self-perceptions of low value and vice versa. As pointed out previously, “The twin sister to loneliness is uselessness” (Russell, 2019), implying that being aware of useful resources play a significant role in interaction with others.

Regarding the R of relationships, the loss of relationships much due to stigma had an impact on the participants’ perceived value. Several of them questioned their value, which increased their insecurities related to establishing new relationships and doubts to whether these relationships could be reciprocal. Did they have anything of value to offer other people? As relationships are considered a cornerstone of human life, not experiencing being of sufficient value to others must be addressed. This is in line with previous research showing that experiencing reciprocal relationships are one of the main struggles among socially excluded young adults with mental illness (Gardner et al., 2019). As per relationships with professionals, not experiencing being treated as a person with value further complicate and function as barriers to recovery processes (Ljungberg et al., 2016; De Ruysscher et al., 2017), and inherently, the promotion of social inclusion.

The lack of validated belonging is evident throughout the 5 Rs. Participants did not only speak of their lack of value as hindering them in experiencing belonging, but also spoke of a perception of modern society’s lack of concern for others. This led some to distancing themselves from others. Few experienced a validated sense of belonging in the mainstream community, though some had better experiences online, others had negative experiences in both areas, and low expectations of others, expecting them to not value them. In this sense, they were worthless in a worthless world. This is interesting when seen in relation to the mattering framework, in that one of its primary goals is to create a more collectivistic way of thinking through valuing, reciprocal relationship between individuals and their surroundings (Prilleltensky and Prilleltensky, 2021). These findings confirm previous studies pointing out the conflicting feelings in attempting to belong or choosing social withdrawal, and the dynamics between belonging and not having a perceived sense of value (Semb et al., 2021).

### Implications

Our findings show that combining the Citizenship and Mattering frameworks might be an applicable framework to promote social inclusion. As previous research has shown, the public mental health and welfare services appear either as facilitators or barriers in promotion of social inclusion depending among other things on the dynamic between the persons and the service providers (Topor et al., 2022; Nord-Baade et al., 2024; Ponce et al., 2016). The messages and actions of service providers contribute to how the individuals perceive themselves.

Regarding implications for practice, the study calls for increased awareness and knowledge on social inclusion in the services, and a need to provide the means to do so. We suggest educating providers on a thorough understanding of the concepts of Citizenship and Mattering, including implications and how these ideas influence young adults with mental illness and complex needs. Once familiar with these topics, providers can engage young adults in discussions, clearly communicating that the aim is to enhance social inclusion in their communities, highlighting the associated benefits, seen in relation to their complex needs and challenges. By outlining the purpose behind social inclusion efforts, providers can help young adults feel valued and empowered to engage in society. We suggest a systematic approach with step-by-step approaches aimed at enhancing the connection to the 5 Rs. This should be employed parallel to exploring and addressing their sense of mattering and relevant means to promote it following person-centered principles.

Future research should investigate this potential and explore how the frameworks can be integrated, including a focus on identity, as neither of the frameworks directly addresses this topic. In addition, studies should explore the service providers’ perspectives and experiences as there are known challenges in implementing this perspective in mental health services (Ponce et al., 2016). Future research should also explore how the concepts of Citizenship and Mattering are understood and experienced by young adults with non-western backgrounds and in non-Western contexts, considering cultural differences and societal norms.

We believe our findings may be an answer to the call for a multifaceted approach and understanding of the paths to social inclusion and exclusion, including a focus on young adults’ subjective experiences and the broader social context and living conditions (Prilleltensky et al., 2023). Further, such a framework may apply to other marginalized age groups and context.

### Strengths and limitations

A team approach was employed in the study with a valuable combination of lived experience, psychological and sociological knowledge. Despite the low number of participants, the people in the target group had many similar experiences and provided rich descriptions, pointing at an acceptable degree of validity in our findings. The study could have benefited from a higher number of participants, but this may not be a major weakness. The participants lived in both rural and urban areas, indicating that the findings might apply independently of demographic context and in similar countries. It should also be noted that none of the participants had immigrant backgrounds. This might constrain the generalizability of the findings to young adults with such a background. In addition, we did not explore team dynamics, available resources or institutional policies that could affect the FACT team and the participant’s experiences. This could have provided additional insights.

## Data Availability

The raw data supporting the conclusions of this article will be made available by the authors, without undue reservation.
